# Optimizing cultivation practices to enhance growth and yield of Indian mustard

**DOI:** 10.1038/s41598-025-95313-2

**Published:** 2025-04-03

**Authors:** Akshita Sharma, Vandna Chhabra, Swati Mehta, Yudhishther Singh Bagal, Rajesh Kumar, Nadhir Al-Ansari, Salah El-Hendawy, Mohamed A. Mattar, Ali Salem

**Affiliations:** 1 Department of Agronomy, School of Agriculture, Lovely Professional University, Phagwara, 144411 Punjab India; 2 Department of Civil, Environmental, and Natural Resources Engineering, Lulea University of Technology, Lulea, 97187 Sweden; 3 Department of Plant Production, College of Food and Agricultural Sciences, King Saud University, P.O. Box 2460, Riyadh, 11451 Saudi Arabia; 4 Department of Agricultural Engineering, College of Food and Agricultural Sciences, King Saud University, P.O. Box 2460, Riyadh, 11451 Saudi Arabia; 5 Structural Diagnostics and Analysis Research Group, Faculty of Engineering and Information Technology, University of Pécs, Pécs, 7622 Hungary; 6 Civil Engineering Department, Faculty of Engineering, Minia University, Minia, 61111 Egypt

**Keywords:** Growth, Mustard, Mulching, Sulphur, Yield attributes, Agroecology, Agroecology

## Abstract

Since mustard is a significant oilseed crop in India, improving cultivation practises is essential for enhancing the productivity. The experiment was laid out in randomized block design with three replications at the farm of Department of Agronomy, Lovely Professional University, Phagwara during the *rabi* season. The work was carried out for two years to analyse the pooled data (2022–2023; 2023–2024) of Indian mustard. A total of 11 treatments were utilized with various sowing techniques, sulphur use, and mulching for enhanced yield on mustard growth and yield traits. With an increase in doses of sulphur, mulching with paddy straw and sowing techniques including flat bed and ridge sowing the growth characteristics such as plant height, number of leaves per plant, number of branches per plant increased and enhanced yield traits. Results from the study revealed that among the various treatments, the application of Treatment T9 = Ridge sowing + recommended NP (100:75 kg/ha) + recommended S (20 kg/ha) + mulching (Paddy straw) had more growth and improved yield as compared to other treatments of Indian mustard. The pooled analysis of data from 2022 to 23 and 2023-24 revealed that treatment T9 achieved the maximum plant heights, measuring 14.8 cm, 80.8 cm, and 131.2 cm at 30, 60, and 90 DAS, number leaves per plant (6.0, 38.8, 83.2) at 30, 60, 90 DAS and number of branches (3.2, 6.7) at 60, 90 DAS. In yield analysis, the greatest number of siliquae per plant (73.6), the longest siliquae length (4.3), seeds per siliquae (23.1), 1000 seed weight peaked at 3.5, seed yield was 1.6 t/ha, stover yield was 4.1 t/ha, and harvest index was 29.6% in 2022-23 analysis. Overall, from pooled analysis the 2022-23 mustard crop had more growth and yield as compared to 2023-24 mustard crop.

## Introduction

Oilseed crops have been grown all over the world and in the agriculture system oilseed sector plays a significant role. *Brassica juncea* is cultivated in the states of Assam, Bihar, Gujarat, Haryana, Himachal Pradesh, Jammu, and Kashmir, Madhya Pradesh, Orissa and Punjab^1^. Cultivation of this oil seed crop is done in tropical as well as temperate regions between October- November, and February-March^2^.

There are three main species (*Brassica juncea*, *Brassica campestris*, and *Brassica napus* L.) grown in the *Indian* subcontinent. In India, about 27.5 million ha area is occupied by oilseeds which represent 14% of the total cropped area with the production of 24.72 million tonnes accounting for 5 per cent of gross national product and 10% value of all agricultural commodities^3^. *Indian* mustard is predominantly cultivated in the states of Rajasthan, Uttar Pradesh, Haryana, Madhya Pradesh, and Gujarat. Rajasthan ranks first in area and production of *Indian* mustard with 2.50 million ha area and 3.71 million tonnes production^2^. In Punjab mustard is grown on 32 thousand hectares with a production of 41.8 thousand tones and productivity of 1306 kg per hectare^4,5^. Flat bed, raised bed, and ridge sowing are some of the ways for shaping the seed bed and land surface. Techniques such as alternating furrow, ridge, linked ridge and furrow sowing are used to increase yields in crops like rapeseed and mustard as compared to typical flatbed sowing^6^. In-ridge sowing, in particular, improves plant growth by boosting soil moisture, facilitating salt leaching and decreasing surface evaporation^7^. Mulching is a typical strategy for reducing evaporation loss from the soil and increasing moisture availability to the crop. Mulching promotes soil moisture, controls soil temperature, inhibits weed development, minimizes nutrient leaching loss, prevents excessive evaporation, lowers soil erosion and improves productivity and quality^8^. Mulch boosted soil organic matter and moisture content while decreasing bulk density and soil strength relative to the control^9,10^. The impacts of mulch on soil temperature, moisture regime, root development and yield are determined by the microclimate, mulch application method and the quality and quantity of mulch materials used. Mulching materials are widely used for the establishment of many herbs and tree species^11^. There are many research studies which showed the positive impacts of mulches on the germination, survival of newly grown plants and transplantation of seedlings and overall performance of crop plants in relation to un-mulched treatments^4,12^.

Sulphur affects important activities either directly or indirectly and is essential to many plant metabolic pathways. The synthesis of amino acids, proteins, lipids and even a component of vitamin A depends on sulphur, which is the fourth major nutrient for plants after nitrogen, phosphorus and potassium^13^. Moreover, sulphur aids in the synthesis of glycosylates (mustard oils), glucosides, enzyme activation, and sulfhydryl (SH-) connections, which give oilseeds their pungency^1,9^. Mustard has the highest Sulphur demand, with an ideal amount of 20 to 60 kg S/ha depending on soil sulphur status and yield potential. Indian mustard responded strongly to sulphur fertilization in oilseeds. Sulphur is essential for the quality and development of seed^14^. Chemical fertilizers used to augment main nutrients are typically inadequate or low in sulphur content^15,16^. The importance of sulphur fertilizer in boosting the production and quality of Indian mustard is becoming more widely acknowledged^17^. However, there is little information available about the ideal level of sulphur, its source, and its effects on mustard seed output and quality. Probably for these reasons, mustard crops require a higher amount of sulphur for optimum growth and development, as well as higher yields^12^. No matter how many other nutrients are added or whether better crop management techniques are used, mustard cannot reach its maximum yield potential on soils lacking in sulphur^14^. Sulphur deficiency is pervasive in India. Due to the expansion of agriculture with high-yielding varieties, sulphur deficiency is becoming more prevalent in Indian soils^18^. Thus, the present study was utilized to evaluate the synergistic effect of the agronomic practices which includes different sowing techniques, sulphur and mulching on the growth and yield of Indian mustard.The purpose of the study aims to identify the effective and sustainable agronomic practices for enhancing the productivity of Indian mustard. Considering Mustard as a key oilseed crop in India, the production challenges faced due to changing climate and low soil fertility this research provides evidence based recommendation for improving crop production and sustainability.

## Materials and methods

**Fig. 1:**
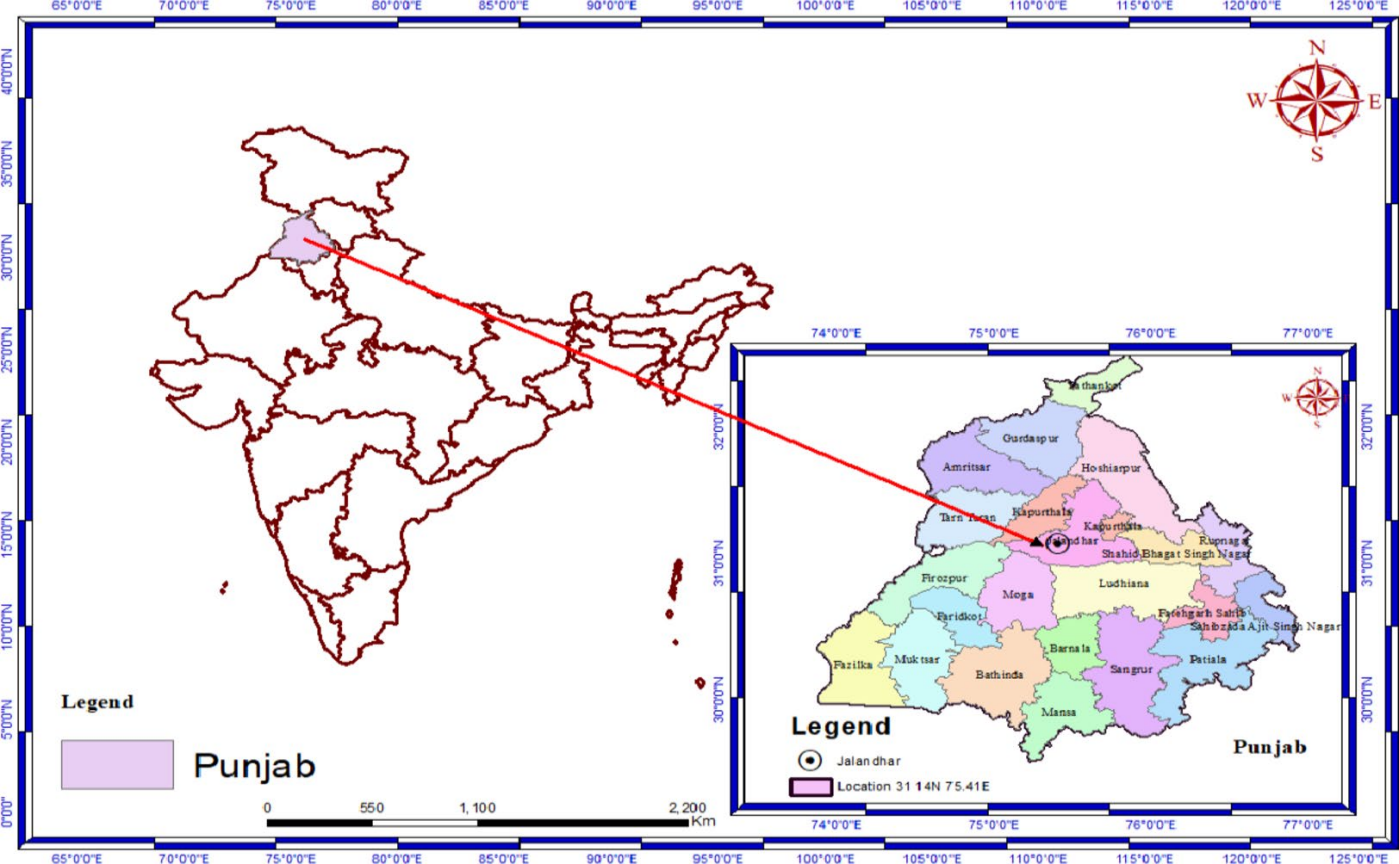
Location of Experimental Site.

The present work was conducted at the field of School of Agriculture, Lovely Professional University, Phagwara, Punjab during winter (*rabi)* season of 2022-23 and 2023-24(2-year data was analysed). Geographically, Lovely Professional University is located at a distance of 8 km from the Phagwara. The experiment site falls in a sub-tropical climate situated at 31 13’28" North latitude and 75 46’ 25" (Fig. 1).

### Climate and weather conditions

**Fig. 2:**
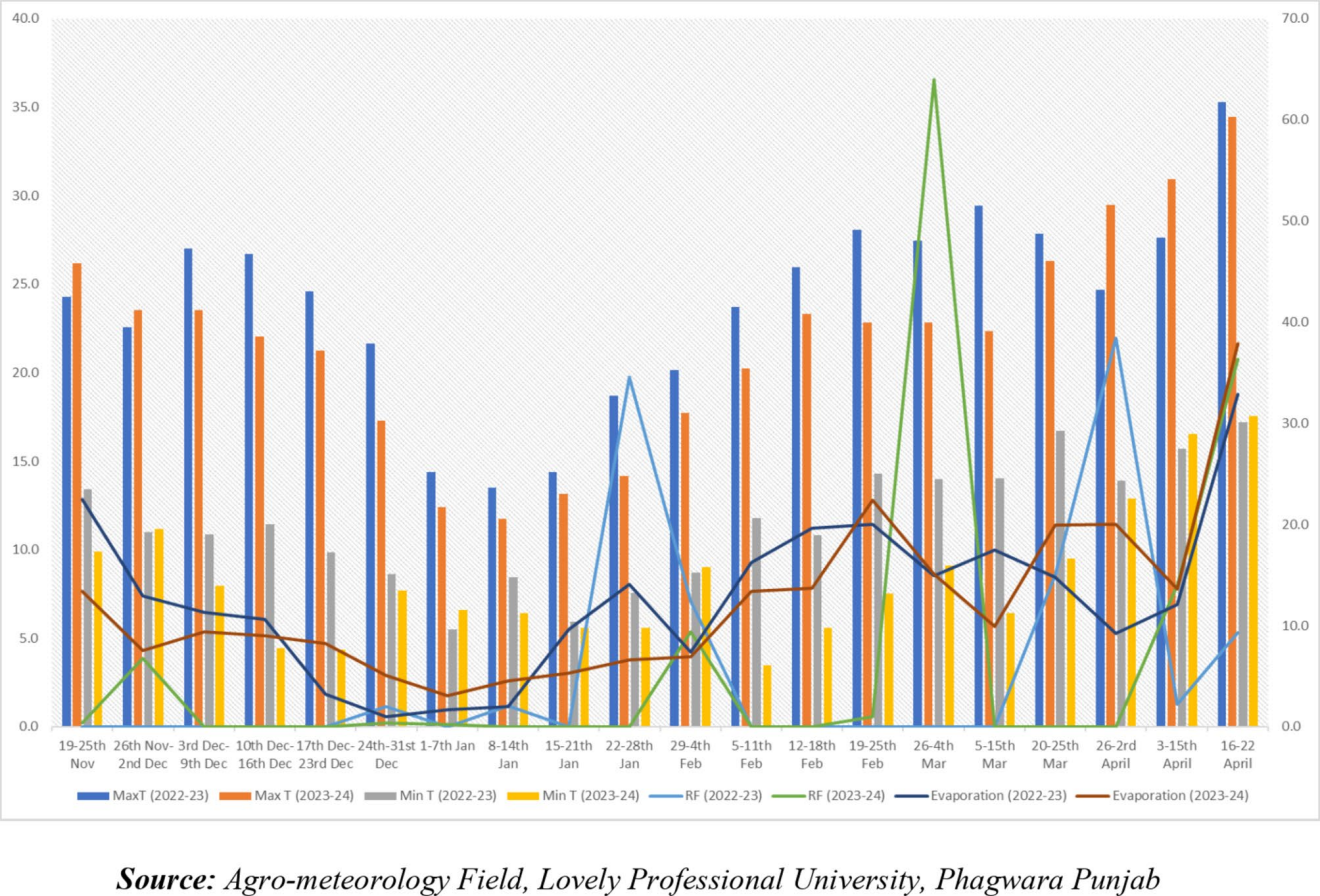
Standard meteorological week data of 2022-23 and 2023-24.

The best climate and weather conditions for Mustard are dry and cool which is summer last (March to June) and winter (November to February). Monsoons from the northeast and southwest both provide rain to Punjab. The monsoon season, which lasts from June to August, is when most rain occurs. Typically, the winter season starts around the end of October and lasts until the end of February. Temperatures drop throughout the first two weeks of November and reach their lowest point in December or January, making those months the coldest of the year. Summer officially starts in mid-February and lasts until the first two weeks of June. May is the hottest month of the season since it is when temperatures begin to increase and reach their peak as shown in (Fig. 2). Four irrigations were given to the crop during the crop seasons.

### Experimental design

The experiment was done in a randomised block design (RBD) which composed of 11 treatments in 3 replications (Table 1). The work was done on Indian mustard for two years (2022-23 and 2023-24) to analyse its pooled data. The plot area was 600 m^2^, with each plot measuring 15 m^2^ and in total 33 plots were included. The crop was sown on November 24 (2022-23) and November 28 (2023-24) with the spacing of 15 × 30 cm followed by line sowing, according to the treatment protocols the recommended doses of Nitrogen and Phosphorus (100:75 kg/ha) were applied in the form of Urea and sulphur (20 kg/ha) in the form of DAP. Thinning was done 25 days after sowing to maintain the optimum population. The crop was harvested in the month of April for both the years.

**Table 1:** Treatment details.

Treatments	Details
T1	Control + Flat sowing
T2	Flat sowing + recommended NP
T3	Flat sowing + recommended NP + recommended S
T4	Flat sowing + recommended NP + 50% S
T5	Ridge sowing + recommended NP
T6	Ridge sowing + recommended NP + recommended S
T7	Ridge sowing + recommended NP + recommended 50% S
T8	Ridge sowing + recommended NP + mulching
T9	Ridge sowing + recommended NP + recommended S + mulching
T10	Ridge sowing + recommended NP + 50%S + mulching
T11	Ridge sowing + recommended 50% NP + 50% S + mulching

### Experimental data

The data for growth and yield attributes of Indian mustard were recorded using three random chosen plants from each treatment after seed sowing. For growth characters’ plant height (cm), number of leaves per plant and number of branches per plant were estimated^19^. The growth parameters are recorded at 30 days intervals.

The yield attributes recorded for the mustard crop are the length of siliquae (cm), number of siliquae per plant, number of seeds per siliquae, test seed weight (g), seed yield (t/ha), stover yield (t/ha), and harvest index (%) (HI *= Economic yield / Total Biological yield* ×100)^20^.

### Statistical analysis

The data collected from the present piece of work at different growth stages were subjected to statistical analysis. The statistical analysis was carried out by using the software OPSTAT version 6.8 and found that most of the parameters considered for this experiment were significant at *p* < 0.05% and also shows a significant difference among the treatments at all the time of observations.

## Results and discussion

### Growth parameters

**Fig. 3:**
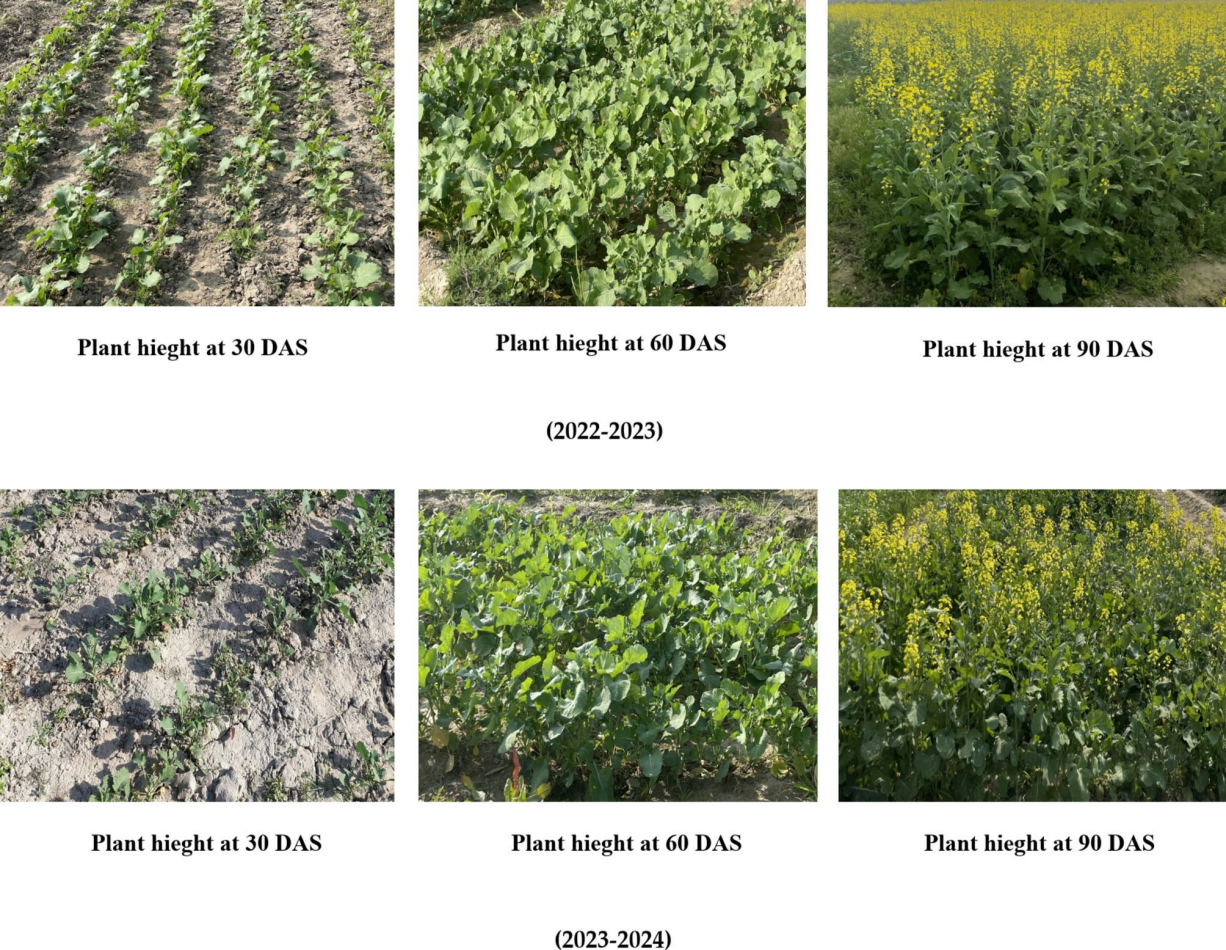
Plant growth at various time intervals (30, 60 and 90 DAS) of 2022-23 and 2023-24 year.

Growth parameters including plant height, number of leaves per plant and number of branches per plant were significantly influenced by different treatments on growth attributes at 30 days intervals (Fig. 3).

#### Periodic plant height

Plant height was recorded at 30, 60, 90 days after sowing (DAS) as presented in (Table 2). In both the 2022-23 and 2023-24 years of experiment, the higher plant height was observed in treatment T9 (Ridge sowing + recommended NP + recommended S + mulching) at 30, 60, and 90 DAS. T9 expanded to be 16.5 cm, 85.3 cm, and 137.7 cm tall in 2022-23 and 13.1 cm, 76.4 cm and 124.8 cm in 2023-24. Treatment T7 and T8 also had significant growth, particularly T9 in 2023-24. Therefore, T9 was statistically at par with T7 and T8 at 60 DAS. The control (T1) had the lower heights in both years.

The pooled analysis of data from 2022 to 23 and 2023-24 revealed that treatment T9 achieved the maximum plant heights, measuring 14.8 cm, 80.8 cm, and 131.2 cm at 30, 60, and 90 DAS respectively. In comparison, the control treatment T1 had the lowest heights, measuring 9.0 cm, 69.8 cm and 122.4 cm periodically.

**Table 2:** Effect of sowing techniques, sulphur and mulching on plant height (cm).

Treatments	Plant height (cm)								
	30 DAS			60 DAS			90 DAS		
	2022-23	2023-24	Pooled	2022-23	2023-24	Pooled	2022-23	2023-24	Pooled
**T1**	9.9	8.1	9.0	74.4	65.3	69.8	125.8	118.9	122.4
**T2**	12.6	10.5	11.6	78.6	74.6	76.6	128.1	123.4	125.7
**T3**	14.3	12.1	13.2	79.0	75.1	77.1	128.4	124.0	126.2
**T4**	14.4	12.4	13.4	79.1	75.6	77.5	128.5	124.3	126.3
**T5**	12.8	12.1	12.5	78.9	75.0	76.9	128.4	123.6	126.0
**T6**	15.1	12.5	13.7	79.4	75.7	77.6	129.5	124.3	126.9
**T7**	15.6	12.5	14.1	81.7	75.7	78.7	129.7	124.5	127.1
**T8**	13.7	12.2	13.0	79.8	75.0	77.4	132.1	123.6	127.8
**T9**	16.5	13.1	14.8	85.3	76.4	80.8	137.7	124.8	131.2
**T10**	13.8	12.5	13.2	80.2	75.8	78.0	132.3	124.5	128.4
**T11**	13.8	12.4	13.1	78.9	74.6	76.4	129.4	123.3	126.4
**SE (d)**	**0.7**	**0.5**	**0.6**	**0.8**	**0.7**	**0.8**	**1.1**	**1.3**	**1.4**
**CD (5%)**	**0.4**	**0.4**	**0.3**	**1.1**	**0.6**	**0.6**	**0.9**	**0.5**	**0.4**

Ridge sowing was significantly superior to flat sowing in increasing plant height as it improved water and nutrient availability, reduced weed competition thus creating favourable atmospheric conditions. Increased plant height is linked to improved cell multiplication, elongation and expression, which are aided by appropriate sulfur availability, resulting in a better nutritional environment throughout active vegetative stages. Statistical investigation shows that both sulfur and mulching have a substantial impact on plant height and branch number. Mulching improves food metabolism, biological activity, photosynthetic pigments, and enzymatic activity, all of which promote vegetative growth. The results are in agreement with the findings of^21,22^ in mustard crop. Similarly, the Sulphur and mulching were utilized on Indian mustard. The results depicted that the application of sulphur @ 40 kg ha^-1^ with paddy straw mulch was found superior over other treatments^23^.

#### Number of leaves per plant

Number of leaves per plant were observed at 30, 60, 90 DAS as detailed in (Table 3). Treatment T9 led to a constant and considerable increase in the number of leaves per plant of Indian mustard throughout both the 2022-23 and 2023-24 growth years and was recorded highest at 7.0, 43.5, 91.6 and 5.0, 34.0, 74.8 at 30, 60, 90 DAS respectively for both years. The data also demonstrate that treatment T9 was statistically equal to treatments T7 and T10, which both performed well, whereas the control treatment T1 had the lowest leaf counts during both the growing seasons.

**Table 3:** Effect of sowing techniques, sulphur and mulching on number leaves per plant.

Treatments	Number of leaves per plant								
	30 DAS			60 DAS			90 DAS		
	2022-23	2023-24	Pooled	2022-23	2023-24	Pooled	2022-23	2023-24	Pooled
**T1**	5.2	4.2	4.7	37.1	31.5	34.3	85.9	73.3	79.6
**T2**	6.2	4.3	5.3	40.6	32.6	36.6	87.5	73.5	80.5
**T3**	6.3	4.4	5.4	41.3	32.7	37.0	88.1	73.9	81.0
**T4**	6.4	4.5	5.5	42.3	33.7	37.9	89.0	74.2	81.6
**T5**	6.3	4.3	5.3	41.2	32.6	36.9	87.9	73.6	80.7
**T6**	6.4	4.5	5.4	41.4	32.7	37.4	88.2	73.9	81.1
**T7**	6.6	4.6	5.6	42.7	33.7	38.2	89.4	74.5	82.9
**T8**	6.5	4.4	5.5	42.1	32.7	37.4	88.1	73.6	80.9
**T9**	7.0	5.0	6.0	43.5	34.0	38.8	91.6	74.8	83.2
**T10**	6.9	4.6	5.7	42.7	33.8	38.3	90.1	74.6	82.3
**T11**	6.8	4.5	5.7	42.6	33.6	38.1	90.0	74.5	82.2
**SE (d)**	**0.1**	**0.1**	**0.1**	**0.3**	**0.1**	**0.0**	**0.2**	**0.1**	**0.2**
**CD (5%)**	**0.1**	**0.1**	**0.1**	**0.6**	**0.3**	**0.4**	**0.5**	**0.2**	**0.3**

From the pooled analysis of data, it was observed that significantly higher value for number leaves per plant (6.0, 38.8, 83.2) at 30, 60, 90 DAS was obtained with the application of Ridge sowing + recommended NP + recommended S + mulching (T9). Lowest number of leaves per plant to the tune of (4.7, 34.3, 79.6) was recorded in treatment T0 at 30, 60, 90 DAS, respectively.

The continuous increase in the number of leaves per plant in treatment T9 is due to the combined impacts of ridge sowing, appropriate sulfur application, and mulching. These variables provide an optimum growing environment by improving root growth, increasing nutrient availability, retaining soil moisture, and limiting weed competition which increases the vegetative growth of the mustard, influencing the number of leaves of mustard crop. Sulphur is important for the synthesis of amino acids and proteins, both of which are required for leaf development. Mulching helps to regulate soil temperature and moisture, supporting healthy root systems and, as a result, improved leaf growth^21,22^. Similar, results are evaluated by^24,25^. Likewise, the effect of mulching and sulphur was evaluated on growth and yield of mustard crop in which the results determined that the application ofPoly sheet mulch + Sulphur 60 kg/ha was foundmore productive with maximum growth^26^.

#### Number of branches per plant

Number of branches per plant recorded at 60, 90 DAS as presented in (Table 4). During the year 2022-23 and 2023-24, the highest number of branches per plant was found with the application of T9 (Ridge sowing + recommended NP + recommended S + mulching) at 60 and 90 (DAS). In 2022-23, T9 had 3.6 branches at 60 DAS and 7.5 at 90 DAS, while in 2023-24, it had 2.8 at 60 DAS and 5.8 at 90 DAS. Treatment T9 was statistically equal to treatments T7 and T10 in both years, while control treatment T1 had the lowest branch numbers. The results show a general tendency of greater branch numbers in 2022-23 compared to 2023-24.

**Table 4:** Effect of sowing techniques, sulphur use and mulching on number of branches per plant.

Treatments	Number of branches per plant					
	60 DAS			90 DAS		
	2022-23	2023-24	Pooled	2022-23	2023-24	Pooled
**T1**	2.3	2.1	2.2	6.4	5.4	5.9
**T2**	2.7	2.4	2.5	6.5	5.5	6.0
**T3**	2.9	2.5	2.7	6.6	5.5	6.1
**T4**	3.1	2.6	2.8	6.8	5.6	6.2
**T5**	2.8	2.4	2.6	6.9	5.5	6.2
**T6**	3.1	2.6	2.8	7.0	5.6	6.3
**T7**	3.4	2.6	3.0	7.4	5.6	6.5
**T8**	3.1	2.5	2.8	7.1	5.5	6.3
**T9**	3.6	2.8	3.2	7.5	5.8	6.7
**T10**	3.1	2.7	2.9	7.1	5.7	6.5
**T11**	3.0	2.5	2.7	6.9	5.5	6.2
**SE (d)**	**0.0**	**0.0**	**0.0**	**0.1**	**0.0**	**0.0**
**CD (5%)**	**0.1**	**0.1**	**0.1**	**0.1**	**0.0**	**0.1**

From the pooled analysis of data, it was observed that significantly higher value for number of branches (3.2, 6.7) at 60, 90 DAS was obtained with the application of treatment T9. Lowest number of branches to the tune of (2.2, 5.9) was received in treatment T1 at 60, 90 DAS, respectively.

By establishing ideal growing circumstances that enable the crop to completely express its genetic potential, ridge sowing, mulching techniques combined with the right amounts of sulfur can maximize the production of Indian mustard. The findings highlight the relevance of integrated fertilizer and moisture management in increasing crop output through improved branching and biomass buildup^24^. An improved environment for mustard growth and development may be the cause of the overall increase in crop growth under the impact of the suggested dose of sulphur application and mulching. Similar, results are reported by^25^ in mustard crop. A reviewer, found the effect of Sulphur and mulching on *Indian* mustard which depicted that the application of sulphur @ 40 kg ha^-1^ with paddy straw mulch was found superior over other treatments^23^.

### Yield attributing characters

Yield attributes including length of siliqua, number of siliquae per plant, number of seeds per siliquae were significantly influenced due to sulphur levels and mulching on yield contributing characters are presented in Table 5.

**Table 5:** Effect of sowing techniques, sulphur and mulching on yield attributing traits.

Treatments	Number of siliquae per plant			Siliquae length (cm)			Number of seeds per siliquae		
	2022-23	2023-24	Pooled	2022-23	2023-24	Pooled	2022-23	2023-24	Pooled
T1	33.1	31.4	32.3	3.1	3.1	3.2	15.1	14.1	14.6
T2	66.5	64.7	65.6	3.6	3.4	3.6	19.6	18.5	19.0
T3	69.0	67.9	68.5	3.9	3.7	3.8	20.8	19.7	20.3
T4	72.1	70.7	71.4	4.1	3.9	4.1	22.2	21.1	21.6
T5	68.7	66.3	67.5	3.7	3.5	3.7	19.8	18.6	19.7
T6	70.4	68.5	69.4	4.0	3.8	3.9	21.7	20.1	20.9
T7	73.7	71.7	72.8	4.2	4.1	4.2	22.4	21.6	22.0
T8	71.2	69.5	70.3	3.9	3.9	3.9	22.7	21.7	22.2
T9	74.6	72.6	73.6	4.4	4.2	4.3	23.9	22.4	23.1
T10	71.7	70.6	71.1	3.8	3.8	3.8	22.5	21.2	21.8
T11	71.1	70.5	70.8	3.8	3.7	3.7	21.9	20.9	21.6
**SE (d)**	**0.3**	**0.4**	**0.3**	**0.0**	**0.0**	**0.0**	**0.2**	**0.3**	**0.2**
**CD (5%)**	**0.7**	**0.7**	**0.6**	**0.1**	**0.1**	**0.1**	**0.4**	**0.5**	**0.4**

#### Siliquae per plant, siliquae length, seeds per siliquae

The investigation of Indian mustard yield attributing characters from 2022 to 24 consistently showed that treatment T9 (Ridge sowing + recommended NP + recommended S + mulching) was statistically superior to all other treatments across all the parameters. T9 had the highest values in 2022-23, with 74.6 siliquae per plant, 4.4 cm siliquae length and 23.9 seeds per siliquae, and followed the same trend in 2023-24, with 72.6 siliquae per plant, 4.2 cm siliquae length, and 22.4 seeds per siliquae. The control treatment T1 consistently performed poorly across both years, with the lowest values for all parameters (33.1, 31.4 siliquae per plant, 3.1, 3.1 cm siliquae length, and 15.1, 14.1 seeds per siliquae) for 2022-23 and 2023-24 respectively. However, T9 wasstatistically at par with different treatments across the parameters with T7 and T10 for siliquae per plant, T7 and T8 for siliquae length, and T7 and T8 for seeds per silique implying that different combinations of these agricultural practices can achieve similar levels of productivity. The overall superior performance in 2022-23 than 2023-24demonstrates the impact of environmental circumstances on crop development.

In addition, in pooled analysis T9 produced the greatest number of siliquae per plant (73.6), the longest siliquae length (4.3) and the more seeds per siliqua (23.1), exceeding all other treatments. In contrast, the control treatment T1 consistently performed poorly across all parameters, with the smallest values for siliquae per plant (32.3), siliquae length (3.2 cm) and seeds per siliqua (14.6).

These findings reveal how well mulching, balanced nutrition, and ridge sowing can maximize yield potential and reproductive development in Indian mustard. It can also be attributed to improved photosynthesis, efficient soil moisture management and ideal nutrient availability. Ridge sowing creates a favourable microenvironment by improving root growth, nutrient and water uptake for mustard crop. Mulching holds moisture and controls soil temperature, nitrogen and sulphur work together to supply vital nutrients that support plant health and productivity, fostering an ideal growing environment^25^. Higher nutrient availability and absorption of nutrients that resulted in translocation of assimilates into crop and hence in return increased seeds per siliquae. Similar, results were evaluated by^26–28^ in Indian mustard crop for siliquae per plant, siliquae length and seeds per siliquae parameters.

### Yield parameters

Yield parameters include test weight (g), seed yield (t/ha), stover yield (t/ha) and harvest index (%) were significantly influenced by sowing techniques, Sulphur application and mulching as presented in (Table 6).

**Table 6:** Effect of sowing techniques, sulphur use and mulching on test weight (g), seed yield (t/ha), Stover yield (t/ha) and harvest index (%).

Treatments	Test weight (g)			Seed yield (t/ha)			Stover yield (t/ha)			Harvest index (%)		
	At harvest											
	2022-23	2023-24	Pooled	2022-23	2023-24	Pooled	2022-23	2023-24	Pooled	2022-23	2023-24	Pooled
**T1**	1.2	1.1	1.2	0.9	0.8	0.8	2.4	2.3	2.4	27.2	26.4	26.8
**T2**	2.9	2.5	2.7	1.3	1.2	1.2	3.1	3.1	3.1	28.8	28.2	28.5
**T3**	3.1	3.0	3.1	1.4	1.3	1.3	3.2	3.2	3.2	28.9	28.4	28.6
**T4**	3.5	3.1	3.3	1.7	1.4	1.4	3.5	3.4	3.5	29.1	28.7	28.9
**T5**	3.0	2.6	2.8	1.3	1.3	1.3	3.3	3.2	3.3	28.9	28.8	28.8
**T6**	3.2	2.8	3.0	1.4	1.3	1.3	3.4	3.3	3.4	29.5	29.0	29.3
**T7**	3.6	3.1	3.4	1.5	1.4	1.4	3.5	3.5	3.5	29.8	29.1	29.5
**T8**	3.3	2.7	3.0	1.5	1.5	1.5	3.6	3.6	3.6	28.5	28.3	28.4
**T9**	3.7	3.2	3.5	1.6	1.6	1.6	4.1	4.0	4.1	29.8	29.4	29.6
**T10**	3.3	3.1	3.2	1.5	1.4	1.4	3.6	3.6	3.7	28.9	28.7	28.8
**T11**	3.7	2.8	3.0	1.4	1.4	1.4	3.6	3.7	3.5	29.0	28.8	28.9
**SE (d)**	**0.3**	**0.2**	**0.3**	**0.1**	**0.1**	**0.2**	**0.2**	**0.3**	**0.4**	**0.6**	**0.7**	**0.7**
**CD (5%)**	**0.1**	**0.1**	**0.1**	**0.0**	**0.0**	**0.0**	**0.1**	**0.1**	**0.1**	**0.5**	**0.7**	**0.5**

### Test weight (g), seed yield (t/ha), Stover yield (t/ha) and harvest index (%)

According to a complete analysis of yield parameters of two growing seasons (2022-23 and 2023-24), treatment T9 (Ridge sowing + recommended NP + recommended S + mulching) consistently significant amongst all analysed parameters. T9 recorded the highest values (3.7 and 3.2 in both years respectively) for 1000 seed weight, exhibiting statistical parity with T7 and T10 in 2022–2023 and with T7 and T10 in 2023–2024. Additionally, T9 treatment boosted seed yield, with yields of 1.6 t/ha and 1.6 t/ha in both growing seasons, which was statistically equal to T8 and T10 in the first year and T8 and T10 in the second. Likewise, stover yield was maximized at T9 treatment in both years at 4.1 t/ha and 4.0 t/ha, respectively, demonstrating statistical parity with T8 and T10. The harvest index peaked under T9 (29.8% and 29.4% in the respective years), and it was statistically at par with T6 and T7 in both the seasons. The control treatment T1 was recorded with the lowest values in all the respective parameters.

Based on the pooled study of both growing seasons (2022-23 and 2023-24), treatment T9surpassed all the yield parameters, with consistently higher values that was seen in than ID=“EN189”>. Under T9 treatment, 1000 seed weight peaked at 3.5, seed yield was 1.6 t/ha, stover yield was 4.1 t/ha, and harvest index was 29.6%. In comparison, the control treatment (T1) consistently had the lowest values for all parameters: 1000 seed weight of 1.2, seed yield of 0.8 t/ha, stover yield of 2.4 t/ha, and harvest index of 26.8%.

The synergistic effect of methods of sowing, sulphur application and mulching are principally responsible for the improved yield parameters under treatment T9. Under these conditions, the increased availability and efficient transmission of photosynthates helped to improve all yield components. Sowing on ridges leads to more light exposure, hence increasing the photosynthetic rate crop which increases the yield of the mustard crop. Sulphur application and mulching improved nutrient availability and utilization, resulting in increased 1000 seed weight^29,30^, increased seed yield through improved economic sink strength in mustard, increased stover yield due to increased leaf area and dry matter accumulation, and an improved harvest index^31,32^. Mulching provided a continuous supply of nutrients and improved moisture conservation, creating ideal circumstances for photosynthate synthesis and translocation, ultimately resulting in greater crop performance across all evaluated parameters. Similar results were reported in Indian mustard crop and the result revealed that application of different levels combination of phosphorus and sulphur fertilizers increased test weight, seed yield, stover yield and harvest index of mustard. It was found that treatment T8 (P_60_ kg/ha^−1^ + S_60_ kg/ha^−1^) had more yield as compared to other treatments^33^.

## Conclusions

In the present investigation, different sowing methods, sulphur use and mulching treatments are tested in terms of their impact on mustard growth and yield attributes. It was concluded from the results that the application of Treatment T9 = Ridge sowing + recommended NP (100:75 kg/ha) + recommended S (20 kg/ha) + mulching (Paddy straw) had more growth and improvement in yield as compared to other treatments of Indian mustard. Overall from pooled analysis, the 2022-23 mustard crop had more growth and yield as compared to 2023-24 mustard crop. This improving effect might possibly be attributed to the nutrient’s favorable influence on metabolism and biological activity, as well as its stimulatory effect on photosynthetic pigments and enzymatic activity, which promotes plant vegetative growth and yield. Thus, it is advised that ridge sowing, sulfur and mulching can be employed to increase the yield components of the mustard crop.

## Data Availability

The datasets used and/or analyzed during the current study are available from the corresponding author upon rea-sonable request.
